# A Recoding Method to Improve the Humoral Immune Response to an HIV DNA Vaccine

**DOI:** 10.1371/journal.pone.0003214

**Published:** 2008-09-15

**Authors:** Yaoxing Huang, Michael Krasnitz, Raul Rabadan, Daniela M. Witten, Yang Song, Arnold J. Levine, David D. Ho, Harlan Robins

**Affiliations:** 1 Aaron Diamond AIDS Research Center, The Rockefeller University, New York, New York, United States of America; 2 Institute for Advanced Study, Princeton, New Jersey, United States of America; 3 Department of Statistics, Stanford University, Stanford, California, United States of America; 4 Computational Biology Group, Fred Hutchinson Cancer Research Center, Seattle, Washington, United States of America; University of California San Francisco, United States of America

## Abstract

This manuscript describes a novel strategy to improve HIV DNA vaccine design. Employing a new information theory based bioinformatic algorithm, we identify a set of nucleotide motifs which are common in the coding region of HIV, but are under-represented in genes that are highly expressed in the human genome. We hypothesize that these motifs contribute to the poor protein expression of *gag, pol,* and *en*v genes from the c-DNAs of HIV clinical isolates. Using this approach and beginning with a codon optimized consensus *gag* gene, we recode the nucleotide sequence so as to remove these motifs without modifying the amino acid sequence. Transfecting the recoded DNA sequence into a human kidney cell line results in doubling the *gag* protein expression level compared to the codon optimized version. We then turn both sequences into DNA vaccines and compare induced antibody response in a murine model. Our sequence, which has the motifs removed, induces a five-fold increase in gag antibody response compared to the codon optimized vaccine.

## Introduction

In the effort to create a vaccine for human immunodeficiency virus type 1 (HIV-1), poor immune response to the HIV proteins is a fundamental problem. For a DNA vaccine, the immune response is correlated with protein expression levels, so an increase in expression of these proteins could alleviate a significant road block to the construction of a viable DNA vaccine.[1], [2] Transcription of the HIV DNA copy is an inefficient process that is aided by the addition of an HIV protein (TAT). In addition the large mRNAs from HIV have inherent RNA processing problems and are not efficiently exported from the nucleus in the absence of a helper protein (REV). These m-RNA synthesis and transport problems are presumably due to a set of RNA sequences or structures encoded in HIV DNA and RNAs.[3], [4] We hypothesize that identifying and removing these signals, which cause the poor synthesis and nuclear confinement of HIV RNA, should significantly increase expression levels of these proteins and improve the immune response.

The genome of HIV-1 contains nine open reading frames (ORFs), all of which are expressed from a single promoter through alternative splicing. The splice forms for the six ORFs Gag, Pol, Env, Vpu, Vif, and Vpr, along with the full length mRNA, contain Rev response elements (RREs) encoded in their RNA. In the absence of the Rev protein, these six ORFs are poorly expressed. The remaining three ORFs, Tat, Rev, and Nef, are expressed efficiently independently of the Rev protein.[3]

The mRNAs which contain RREs likely also contain an as yet unidentified signal or set of signals which prevents normal expression.[4] A primary cause of the poor expression of these ORFs is nuclear confinement.[3] The genome of HIV-1 has an anomalous nucleotide distribution as compared with the set of known coding genes in the human genome. Only 314 of the approximately 25000 genes in the human genome have a higher percentage of adenine (A) than the average clinical isolate of HIV-1. Similar A content can be found in other retrotranscribing viruses (e.g. LINE elements, lentiviruses, spumaviruses); this suggests that retroviruses undergo different selection pressures than ones directing the evolution of the human genome. In the early 90's, the Pavlakis lab showed experimentally that synonymous changes to the Gag ORF, which decrease the A content, significantly increase expression of Gag in human cells.[4] Codon-optimized strains, which are widely used in present experiments and vaccine trials, can increase the protein expression level of Gag transfected into human cells between 500 and 1000 fold.[1], [2] However, the substantial increase in expression due to codon optimization can, at best, indirectly address the problem of poor synthesis and nuclear isolation. We identify multiple nucleotide motifs from a systematic comparison of the HIV-1 genome and the human genome, which we conjecture to play a causative role in poor synthesis and nuclear confinement.

In this study, the short motif, AGG, is found to have the maximal differential representation between the coding genes in the human genome and the HIV-1 genome. This identification was made through the use of an information theoretic motif-finding method called the Robins-Krasnitz algorithm, described previously[5].The algorithm identifies dozens of motifs that exhibit substantial differences in representation between the HIV-1 genome and the human coding genes. The study presented here focuses on a single motif in order to isolate its contribution to expression level in a controlled experiment. A codon-optimized version of HIV-1 consensus *gag* is modified, making synonymous changes to reduce the number of occurrences of AGG. Two plasmids are constructed, one with the original codon-optimized (CO) sequence of *gag* (AD-gag) and the other with the motif-optimized (MO) sequence with AGG significantly reduced (RK-gag). The (DNA) constructs are transfected into a human epithelial cell line (293 cells) and expression of Gag is shown to be 70% higher for the MO sequence. The two sequences of *gag* are also tested as DNA vaccines in a murine model for differential immune responses between the two constructs. The mice with the MO version of the vaccine have a 4.5-fold greater anti-Gag antibody response after 4 weeks. With a DNA boost at four weeks and a second readout of anti-Gag antibody titers measured at six weeks, the gap continues to widen between the MO and CO vaccines to 6-fold.

## Results

### Finding the signal and recoding Gag

The Robins-Krasnitz algorithm finds short nucleotide motifs in coding regions of the human genome that are independent of amino acid order and codon usage.[5] Codon usage is defined as the distribution of synonymous codons present in a given gene. The result of the Robins-Krasnitz algorithm is a set of exact nucleotide motifs of length 2–7 bases which are under and over represented in the coding regions of the human genome. The frequency of these motifs in the HIV genome can then be assessed. See Methods for details.

Beginning with the set of the 100 most under- and over-represented motifs in the human genome, our study attempts to identify the motif with the largest density difference between the HIV genome and the human genome, after accounting for A content. The motifs are restricted to the set of human genes with A content within 1% of the average HIV A content. The ratios of the densities in the HIV genome are then divided by densities in the human coding regions. If the human density is greater than that of HIV, the quantity is replaced by its reciprocal. We predict that the motif with the largest ratio of densities is responsible for nuclear isolation of HIV mRNAs.

The triplet AGG, which is significantly under represented in the coding region of the human genome, is found with a high frequency in HIV when the nucleotide bias of HIV is taken into consideration. We hypothesize that recoding the ORFs of HIV by reducing the frequency of the motif AGG will increase protein expression.

For this initial study, our experimental tests focused on the Gag gene. The codon-optimized sequence of *gag*, referred to as AD-gag, is recoded by systematically removing all AGGs such that the amino acid sequence is not modified and very rare codons are not introduced. The result is the motif optimized RK-gag. Both the AD-gag and RK-gag sequences are found in the supplementary materials.

### Testing expression

**Figure 1:**
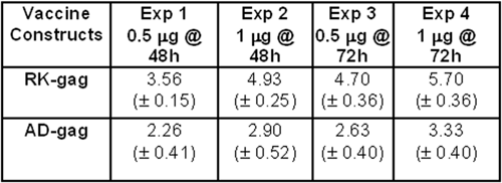
Gag expression in transiently transfected 293 cells. Figure 1 presents the results from four independent transfection experiments. The results are expressed as the mean P24 value (ng/ml, ±SD) of triplicates. The two different *gag* sequences are the codon optimized version (AD) and the motif optimized version (RK) that we created. Our (RK) version of the Gag gene has approximately two-fold higher expression than the codon optimized version.

First, we determine whether our RK-gag has increased expression as compared to the codon optimized version, AD-gag. Since our version of Gag is undoing part of the codon optimization present in the AD-gag sequence, the protein expression levels should be expected to decrease unless the motif AGG significantly inhibits mRNA synthesis or processing or transport. To compare expression levels, human 293 cells were transfected *in vitro* with one of the two different versions of Gag (see methods for details). Gag protein expression was measured in the extracts of transfected cells by a quantitative P24 ELISA. RK-gag was 70% higher than the codon optimized AD-gag, with a p-value <0.00001 calculated by a permutation test (see Figure 1).

### Humoral immune response

**Figure 2:**
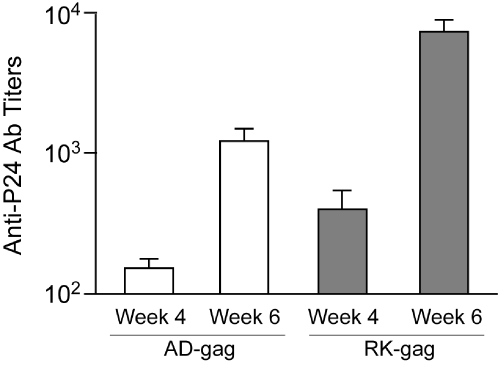
Immunogenicity of Gag DNA vaccines in mouse. The two different versions of Gag were made into DNA vaccines and injected into Balb/C mice with 25 µg/dose, then boosted at four weeks. Anti-Gag antibody levels were measured by ELISA at the four week and six week time points. The results are expressed as the geometric mean antibody titers (±SD) of each group. RK-Gag, induced an immune response that was five times larger than the codon optimized version at four weeks, which increased to a factor of 6 difference after six weeks.

To test the effect of the almost two-fold gain in expression on the immune response, we created DNA vaccines from each of the sequences. These DNA vaccines were injected into the hind leg muscle of Balb/C mice. The mice were given a booster shot after four weeks. Anti-Gag antibody titers were measured by anti-P24 ELISA at the four week and six week time points (see Methods for details). The results are found in Figure 2. The 70% increase in the expression of the GAG protein *in vitro* translated into more than a five-fold difference in humoral immune response in a mouse model.

Two weeks post-boost, mice were sacrificed and splenocytes were prepared for measuring Gag-specific cell-mediated immune (CMI) responses by an IFN-γ ELISpot assay. Although the difference between these two groups was not statistically significant, there was a trend for RK-gag immunized mice to have stronger CMI responses to both Gag-specific CD4 and CD8 peptides tested (data not shown).

## Discussion

Recoding the Gag gene in order to reduce the occurrences of a single triplet, AGG, substantially improves immune response to an HIV DNA vaccine in a mouse model. This short sequence motif occurs less frequently in the human coding sequence than in the mouse coding sequence by about twenty percent, so it is possible that these results would be even more dramatic in humans. A set of additional steps would be required to move in the direction of a clinically viable vaccine. These include a recoding of the ENV ORF and a test of its ability to induce an improved immune response. Testing these concepts in primates would be a useful step. The goal of this study was to provide convincing evidence that recoding the HIV ORFs can improve HIV protein expression and the immune response compared to the present codon optimization schemes. It likely that including the other motifs identified by the Robins-Krasnitz algorithm in a systematic way has the potential to improve upon the large gains displayed in this study.

Finally this study brings up the question of why the HIV DNA sequence has been selected to express poorly in primate cells, only increasing its levels with the aid of additional proteins that recognize nucleic acid sequences in the genome. Several other retroviruses and retrotransposons have similar sequence complexities. This may reflect an optimal way to regulate these viruses and enhance the viral titers over an extended length of infection. In any event it is becoming clear that nucleotide sequence motifs, in addition to the choice of codons, can have dramatic impacts upon gene expression, RNA processing and transport in a cell.

## Methods

### Robins-Krasnitz algorithm

**Figure 3:**
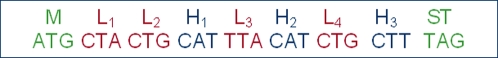
Example of shuffling procedure. The procedure to get the maximal entropy distribution (MED) involves a set of randomized iterations. The triplets of nucleotides coding for each amino acid are permuted randomly among themselves. This is an illustrative example of a mock short protein with eight amino acids. The shuffling procedure randomly permutes L_1_, L_2_, L_3_, and L_4_ and separately permutes H_1_, H_2_, and H_3_. Each iteration produces a new sequence. For this example, there are 12 different combinations for the leucines and three combinations for the histidines resulting in 36 unique sequences. They are weighted in the shuffling procedure so that the MED is attained in the limit of a large number of iterations.

The first step in the algorithm is the creation of a background sequence to compare with the human genome. This background is a completely randomized version of the coding sequences from the human genome subject to the constraints of amino acid order and codon usage in each gene. We design a Monte Carlo program that randomly permutes the codons for each amino acid within each gene. Figure 3 is an illustrative example.

The shuffling procedure described above yields a set of randomized sequences. From these sequences, we need to extract a probability distribution. As long as the number of occurrences of each motif found in the total set of sequences is reasonably large, we can form a probability distribution, estimating the probability of a given motif by its fraction in the set of all motifs.

After the shuffling procedure we can define two distributions, the real distribution found from the actual sequence and the Maximal Entropy Distribution (MED) which we use as the surrogate for the background. We now need a method for choosing under and over-represented motifs. The standard we used is from information theory. The motif that contributes the most bits of information to the difference between the real distribution and the MED is the first motif we chose. Using information theory has the nice feature of putting all results in the same units, number of bits. This allows us to compare motifs of different lengths and motifs that are either over or under-represented. The formula we employ to compute the motif contributing the most bits of information between the two distributions is the Kullback-Leibler distance or the Relative Entropy. We compute the Relative Entropy contribution for each motif and pick out the one with the largest value.

Once we have found the most under- or over-represented motif in the sequence, we have to pick out the motif which is the next most under- or over-represented. However, we cannot simply take the motif which has the next largest Relative Entropy. This is because the motifs are overlapping, so under or over representation of a given motif affects the distribution of all the other motifs. The example of CpG illustrates this point. In the human genome, the dinucleotide motif CG will have the largest Relative Entropy. However, all eight trimers which contain CG fall within the top 50 highest Relative Entropy motifs. This is simply an artifact of the selection against CG. We are required to first remove the contribution of CG from the MED before recalculating the Relative Entropy to find the next motif. If we call the motif *w*, we rescale all motifs that contain *w* by the same amount so that the rescaled MED had the same distribution for *w* as the real distribution. This forces the Relative Entropy of *w* to zero and, at the same time, removes the contribution of *w* from all other motifs. We can readily show that this choice of rescaling monotonically decreases the overall Relative Entropy between the distributions.

The procedure is iterated, so that we remove the contribution of one motif at a time from the Relative Entropy through rescaling of the MED. Then, we choose the next motif. We continue to iterate the procedure, and find additional motifs, until the motif with the largest remaining Relative Entropy is not statistically significant, as determined by comparing shuffled genomes.

### Experimental protocols

HIV-1 subtype B *gag* consensus sequence was obtained from the Los Alamos HIV database (www.hiv.lanl.gov). The complete sequence of parental consensus *gag* was codon optimized to reflect the codon characteristics of eukaryotic expression systems (AD-gag) and assembled in house using overlapping PCR.[4], [6] RK-gag was synthesized by BlueHeron Biotechnology (www.blueheronbio.com). Both constructs have an identical “Kozak signal” located immediately upstream of the initial ATG and were cloned into NotI and XbaI cloning sites of pVAX1 (Invitrogen).

Plasmid DNAs were prepared by GenElute Endotoxin-free plasmid purification system (Sigma). For valuation of *gag* expression *in vitro*, multiple batches of plasmid DNA were prepared to ensure the reproducibility of each of the independent transfection experiments. Briefly, 0.5 and 1 µg of DNA were transfected into 293 cells using the Lipofectamine reagent in a 24-well plate format according to the manufacturer's specification (Invitrogen). Cell culture supernatants were collected at 48 or 72 hours post transfection. Gag expression was measured by a commercial ELISA kit that detects and quantifies P24 in supernatant (PerkinElmer).

For assessing immunogenicity in mouse model, DNA was eluted into saline at the concentration of 0.5 µg/µl. Independent batches of DNA were prepared for immunizations. Six to eight week old female BALB/c mice (Charles River Laboratories) were housed and treated at the Laboratory Animal Research Center of The Rockefeller University in accordance with Institutional Animal Care and Use Committee guidelines. Groups of mice (4 to 5 per group) were immunized with a 25 µg of plasmid DNA vaccine in 50 µl of saline at week 0 and week 4. Serum samples were collected from individual mice at week 4 (4 weeks post first vaccination) and week 6 (2 weeks post second vaccination). Direct ELISA was used to measure serum anti-Gag antibody titers from immunized mice. Briefly, 96-well plates coated with 0.25 µg recombinant P24 protein overnight were blocked for 2 hours with PBS-T containing 5% dry milk and 0.5% BSA. Individual mouse serum samples were added in serial dilutions and incubated for 2 hours. The plates were washed five times with PBS-T and incubated for one hour with AKP-conjugated rat anti-mouse secondary antibodies. The plates were then washed six times with AMPAK washing solution, developed with AMPAK kit (DAKO Corporation). The plates were read on an ELISA reader at 490 nm. The end-point antibody titers were calculated as the reciprocal dilution of the last dilution that was at least 2-fold higher than normal mice sera controls and yields an absorbance of >0.1.

## References

[1] Liu MA, Wahren B, Karlsson Hedestam GB (2006). DNA vaccines: recent developments and future possibilities.. Hum Gene Ther.

[2] Laddy DJ, Weiner DB (2006). From plasmids to protection: a review of DNA vaccines against infectious diseases.. Int Rev Immunol.

[3] Cullen BR (2003). Nuclear mRNA export: insights from virology.. Trends Biochem Sci.

[4] Schneider R, Campbell M, Nasioulas G, Felber BK, Pavlakis GN (1997). Inactivation of the human immunodeficiency virus type 1 inhibitory elements allows Rev-independent expression of Gag and Gag/protease and particle formation.. J Virol.

[5] Robins H, Krasnitz M, Barak H, Levine AJ (2005). A relative-entropy algorithm for genomic fingerprinting captures host-phage similarities.. J Bacteriol.

[6] Kotsopoulou E, Kim VN, Kingsman AJ, Kingsman SM, Mitrophanous KA (2000). A Rev-independent human immunodeficiency virus type 1 (HIV-1)-based vector that exploits a codon-optimized HIV-1 gag-pol gene.. J Virol.

